# Analysis of factors affecting Canadian medical students’ success in the residency match

**DOI:** 10.36834/cmej.68981

**Published:** 2020-07-15

**Authors:** Joshua Lakoff, Kelly Howse, Nicholas Cofie, Sylvia Heeneman, Nancy Dalgarno

**Affiliations:** 1 Queen’s University, Ontario, Canada; 2 Maastricht University, Limburg, Netherlands

## Abstract

**Background:**

In North America, there is limited data to support deliberate application strategies for post-graduate residency training. There is significant interest in determining what factors play a role in Canadian medical graduate (CMG) matching to their first choice discipline and heightened concern about the number of students going unmatched altogether.

**Methods:**

We analyzed matching outcomes of CMGs based on seven years (2013-2019) of residency application data (*n*= 13,499) from the Canadian Residency Matching Service (CaRMS) database using descriptive and binary logistic regression modeling techniques.

**Results:**

The sample was 54% female, with 60% between the ages of 26 and 29, and 60% attended medical schools in Ontario. Applicants who received more rankings from residency programs were more likely (OR = 1.185, *p* < 0.001) to match. Higher research activities (OR = 0.985, *p* < 0.001) and number of applications submitted (OR = 0.920, *p* < 0.001) were associated with a reduced likelihood of matching. Number of volunteer activities and self-report publications did not significantly affect matching. Being male (OR = 0.799, *p* < 0.05) aged <25 (OR = 0.756, *p* < 0.05), and from Eastern (OR = 0.497, *p* < 0.01), or Western (OR = 0.450, *p* < 0.001) Canadian medical schools were predictors of remaining unmatched.

**Conclusions:**

This study identified several significant associations of demographic and application factors that affected matching outcomes. The results will help to better inform medical student application strategies and highlight possible biases in the selection process.

## Abstract (fr)

**Contexte:**

En Amérique du Nord, les données qui soutiennent les stratégies délibérées de candidature pour la formation en résidence postdoctorale sont limitées. Il existe un intérêt important dans la détermination des facteurs qui jouent un rôle dans le jumelage des diplômés canadiens en médecine (DCM) à leur discipline de premier choix et dans les préoccupations croissantes au sujet du nombre d’étudiants non jumelés.

**Méthodes:**

Nous avons analysé les résultats des DCM sur une période de sept ans (2013-2019) à partir de données de demandes de résidence (n = 13 499) tirées de la base de données du Service canadien de jumelage des résidents (CaRMS) en utilisant des techniques de modélisation par régression logistique binaire.

**Résultats:**

L’échantillon comportait 54 % de femmes, avec 56 % âgées de 26 à 29 ans, et 60 % qui allaient à des facultés de médecine en Ontario. Les candidats qui avaient reçu plus de classements des programmes de résidences étaient plus susceptibles d’être jumelés (RC = 1,185, *p* < 0,001). Les activités scientifiques de pointe (RC = 0,985, *p* < 0,001) et un certain nombre de candidatures soumises (RC = 0,90, *p* < 0,001) étaient associées avec une probabilité réduite de jumelage. Un certain nombre d’activités bénévoles et des publications autodéclarées ne modifiaient pas le jumelage de manière importante. Être un homme (RC = 0,799, *p* < 0,05) âgé de moins de 25 ans (RC = 0,756, *p* < 0,05) et de facultés canadiennes de médecine de l’Est (RC = 0,497, *p* < 0,01) et de l’Ouest (RC = 0,450, *p* < 0,001) étaient des prédicteurs des candidatures non jumelées.

**Conclusions:**

Cette étude a établi plusieurs associations importantes de facteurs démographiques et de candidatures qui touchaient les résultats des jumelages. Les résultats aideront à mieux informer les stratégies de candidatures des étudiants en médecine et montrent les biais possibles dans le processus de sélection.

## Introduction

In Canada, the process of graduating from medical school, choosing a preferred discipline and moving on to residency training can be a stressful and costly experience. Nonetheless, each year the vast majority of Canadian medical graduates (CMGs) successfully match to a residency training program through the Canadian Resident Matching Service (CaRMS).^1^ Medical students wishing to pursue postgraduate medical education in Canada apply for programs through CaRMS, a national, independent, not-for-profit, fee-for-service organization.^1^ Applicants submit applications to programs of their choice and may be selected for an interview. Following the national interview period, applicants rank their preferred programs and programs rank their preferred applicants. The final match is determined through a sophisticated computerized match algorithm. Students unsuccessful in matching in the first iteration have an opportunity to participate in a similar second iteration of the match, where they may apply to remaining unfilled training positions.

Out of approximately 3000 current year CMGs (those applying to CaRMS for the first time), there is a small but substantial number of students who remain unmatched after the first and second iterations of the matching process. This number has increased steadily from 11 in 2009, to 46 in 2016 and declined to 31 in 2019.^2^^–^^4^ Projections indicate that by 2021, the number of current year unmatched CMGs will exceed 140 while prior year unmatched CMGs (those entering the match after failing to secure a position in prior attempts) will exceed 190.^2^ In the 2019 iteration of the match, however, due to an additional number of available residency spots, there was a considerable reduction in the number of unmatched CMGs, with only 31 remaining unmatched after first and second iterations (2020 match data were not available at the time of this publication).^1^ Going unmatched may have a considerable impact on a student’s career trajectory as they may be required to continue their medical training, choose another residency discipline or leave the medical profession altogether.

Contributing to this challenging match process is the increased level of competition to secure a residency position resulting from fewer positions available per applicant over time, and a lack of clear selection committee expectations.^5^^,^^6^ This has had negative consequences for students, not only related to stress and uncertainty, but also financially, as students submit more applications in an attempt to improve their probability of matching.^7^^–^^9^ Selection committees are faced with the increased burden of sorting through and interviewing an increasing number of applicants. There is a compounding effect of unmatched students who add to the level of competition in future iterations of the residency match. Limited, and often anecdotal, information is available to support career advisors in providing meaningful guidance for students based on residency match outcomes.

Until recently, prior research on postgraduate residency training admittance has paid less attention to issues directly affecting students’ matching outcomes.^2^^,^^10^^,^^11^ The majority of the extant research on postgraduate residency training comes from the United States and has disproportionately focused on decision making models and research that emphasizes how systemic and individual factors influence medical students’ career choices.^12^^–^^17^ While this body of research is important, it fails to investigate implicit and explicit discipline and program requirements for residency matching, and how these interact with various applicant factors. A few of these studies that have directly examined medical students’ matching outcomes reveal that applicants who get matched to their top choice discipline tend to have higher United States Medical Licensing Examination (USMLE) Step I scores and have more research products including abstracts, posters, and publications.^11^ Other studies, however, show no association of research products with matching.^18^^,^^19^ Survey data from residency program directors also suggest that research is not considered to be of high importance in applicant selection.^20^^–^^22^ Having additional degrees, including a PhD has not been shown to be associated with matching success.^11^ Some studies indicate that applicants who were successfully matched had higher than average quality reference letters, publications, and USMLE scores.^23^^,^^24^ Such applicants were further required to meet subjective criteria relating to maturity, leadership qualities, and interest in academics. In another study among orthopedic residency applicants, Camp et al. (2016) found that completing a rotation in a program increased residents’ chances of matching to that specific program^10^. In a retrospective cohort study of 1,976 applicants to United States anesthesia programs, being female, younger, and having higher USMLE Step 2 scores were found to be associated with matching success, whereas research products did not influence matching.^18^ Similar and comparable findings, although in an American context, have also been reported by studies focusing on other more highly competitive disciplines in the United States.^25^^–^^29^ While these studies explored medical students’ matching outcomes, scholarship on the subject comes primarily from the United States. Findings from these studies may not necessarily be generalizable to the Canadian context since residency training requirements vary across settings and disciplines. Additionally, many of the existing studies on residency matching outcomes have only examined one or a few discipline areas with relatively smaller samples which may, in turn, limit the validity and generalizability of their findings.^18^^,^^19^^,^^21^

Our study focuses on how student demographic and application factors interact with discipline competitiveness to impact residency matching outcomes. These insights will help guide current and future medical students with their career planning and strategies used to achieve successful residency matching outcomes (such as being accepted in their first choice discipline and avoidance of going unmatched). The purpose of this study is to address the following research questions: 1) What are the factors that influence a CMG applicant’s chance of successfully matching to their first choice discipline? and how do these factors vary by discipline competitiveness and year of the match? and 2) What factors influence a CMG applicant’s chance of going unmatched after the second iteration of the match?

## Methods

### Data

This is a cross-sectional study based on anonymous data on CMGs’ residency application outcomes and background factors from the CaRMS database between 2013 and 2019—a large longitudinal dataset comprising of all 30 direct entry disciplines. Current year graduates refer to those students who applied to CaRMS in the year of their graduation from medical school, in contrast to prior year graduates who are generally applying to CaRMS again after failing to secure a spot in a previous year’s match. International medical graduates’ match data were excluded, as the matching logistics differs considerably from the CMGs. The overall sample for the seven-year data is 20,061. After exclusion of missing data (*n* = 6562) resulting from non-response, the final analytic sample (*n* = 13,499) included only applicants with complete information on all the variables of interest. In particular, applicants who had missing data arising from non-response, on variables such as volunteering activities, research activities, and publication output were excluded from the analysis. The study protocol was approved by the Queen’s University Health Sciences Centre and Affiliated Teaching Hospitals Research Ethics Board. The protocol was also reviewed and approved by CaRMS’ data services department before the data were released in accordance with their privacy policy.

### Outcome variables

To address the purpose of the study, we analyzed three binary outcome variables. The first outcome is a measure of whether an applicant matched to their first choice discipline (as determined by their highest ranked discipline) in the first iteration of the match. The second outcome measures whether an applicant matched to any choice of discipline in the first iteration only. The third outcome measures whether they matched to any choice of discipline in both the first and second iterations.

### Independent variables

The independent variables included in the analyses focus on factors that may influence students’ matching success or failure. We assessed the effects of factors such as: number of applications submitted by the applicant; self-reported volunteer activities, research activities and number of publications; and the number of schools ranking a student on matching outcomes. Guided by prior research, we controlled for demographic factors including age, gender, year of the match, and region of medical school attended. Regions were stratified as Western (British Columbia, Alberta, Saskatchewan, and Manitoba), Ontario, Quebec, and Eastern (Nova Scotia and Newfoundland). Guided by the approach by Scott et al. (2012), the analysis was stratified by the degree of discipline competitiveness.^15^ As per CaRMS supply and demand data, we defined and operationalized competitive programs as those whose ratio of the number of available first iteration discipline positions (supply) to the number of applications received (demand) was less than one. Conversely, programs with a computed ratio of greater than or equal to one were classified as less competitive (see Table 5 in Appendix A).

### Analytical strategy

We employed descriptive statistical techniques to define and describe the characteristics of the analytic sample and examined the proportion of students who matched to their first and other choice disciplines in the first and second iterations of the match. Given the dichotomous nature of the main outcome variables of interest, we estimated a series of binary logistic regression models and assessed the main effects of all independent variables on applicants’ likelihood of successfully matching to a first choice of discipline.^30^^–^^32^ For a more intuitive understanding, the logit coefficients were transformed into odds ratios. An odds ratio greater than one indicates that applicants with a particular attribute have a higher likelihood of matching to a chosen discipline. If the ratio is less than one, there is a lower likelihood. STATA 14 was used to analyze the data.

## Results

The descriptive statistics are presented in Table 1. The sample was 54% female with the majority being between aged 26 to 29 years (60%) and having attended medical schools located in the province of Ontario (60%). Ninety-seven percent of applicants were current year graduates.

**Table 1:** Descriptive statistics of all variables used in the analyses

	Overall sample characteristics			Competitive Programs	Less competitive Programs
	Mean	SD	Percent (%)	Mean	Mean
Number of applications submitted	19.49	12.73		18.25	20.32
Number of volunteering activities	7.94	5.41		8.02	7.89
Number of research activities	8.16	6.73		8.83	7.70
Number of publications	8.60	10.37		9.82	7.76
Number of program ranks received	11.21	7.97		9.18	12.59
Age (in years)				%	%
<=25			22.13	24.77	20.33
26-29			60.00	59.53	60.32
30-34			14.97	13.63	15.88
35+			2.90	2.07	3.47
Gender					
Female			55.29	49.43	56.49
Male			44.71	50.57	43.51
Location of medical school					
Ontario			41.17	39.50	42.31
West			33.04	32.58	33.35
Quebec			18.00	19.27	17.14
East			7.79	8.65	7.20
Current year graduate					
No			2.63	3.37	67.87
Yes			97.37	96.63	2.13
Program competitiviness					
Competitive			40.43		
Less competitive			59.57		
Matched to first choice in first iteration					
No			16.24	18.76	14.53
Yes			83.76	81.24	85.47
Matched to any choice in first iteration					
No			6.03	14.91	0.00
Yes			93.97	85.09	100.00
Matched to any choice in first and second iterations					
No			3.38	8.35	0.00
Yes			96.62	91.65	100.00
N	13499	13499	13499	5458	8041

Almost 60% of the sample applied to less competitive disciplines, defined above. On average, applicants submitted approximately 19.5 applications and were ranked by an average of 11.2 programs. Applicants had a self-reported average of 7.9 volunteering activities, 8.2 research products, and 8.6 publications. Approximately 94% of the sample matched to any choice of discipline in the first iteration, with 84% matching to their first choice discipline. At the end of both the first and second iterations, 97% of students were matched to any choice of discipline. Applying to a less competitive discipline as one’s first choice discipline had a 100% match success rate after the first iteration of the match, with an 85% likelihood of matching to the first choice discipline. Applying to a competitive discipline as one’s first choice discipline had only an 85% success rate of matching to any discipline in the first iteration, with an 81% likelihood of matching to the first choice discipline, and 8% remaining unmatched after both the first and second iterations.

Multivariate logistic regression results examining the odds of matching to a first choice discipline in the first iteration of the match are shown in Table 2. Results from the full sample suggest that the number of applications submitted, number of research activities, number of times ranked by programs, and region of medical school attended are significantly associated with the odds of being matched to a first choice discipline. Applicants who submitted a relatively higher number of applications (OR = 0.920, *p* < 0.001) and who engaged in more research activities (OR = 0.985, *p* < 0.001) are significantly less likely to match to their first choice discipline in the first iteration of the match. This means that for every additional application submitted, the odds of matching to a first choice discipline in the first iteration decreases marginally by 8%. Similarly, the odds of matching to a first choice discipline decreases marginally by less than 2% for applicants who were involved in more research activities. Compared to applicants who graduated from a medical school in Ontario, graduates from Quebec (OR = 0.481, *p* < 0.001) are approximately 52% less likely to match to their first choice discipline. Applicants who received more rankings by programs (OR = 1.185, *p* < 0.001) are almost 19% more likely to match to their first choice discipline.

**Table 2:** Logistic regression models examining the likelihood of matching to a first choice discipline in the first iteration

	Overall sample Model 1			Competitive programs			Less competitive programs Model 3		
	Odds ratio (OR)	Confidence interval		Odds ratio (OR)	Confidence interva		Odds ratio (OR)	Confidence interval	
Number of applications submitted	0.920***	0.915	0.926	0.923***	0.912	0.933	0.921***	0.914	0.928
	(0.003)			(0.005)			(0.004)		
Number of volunteering activities	0.991	0.982	1.000	0.999	0.984	1.015	0.988	0.976	1.000
	(0.005)			(0.008)			(0.006)		
Number of research activities	0.985***	0.978	0.992	0.982**	0.970	0.993	0.982***	0.972	0.992
	(0.004)			(0.006)			(0.005)		
Number of publications	1.001	0.996	1.006	1.004	0.996	1.011	0.993	0.987	1.000
	(0.003)			(0.004)			(0.004)		
Number of program ranks received	1.185***	1.171	1.199	1.470***	1.430	1.511	1.120***	1.105	1.135
	(0.007)			(0.021)			(0.008)		
Age (ref.: 26-29 years)									
< 25	1.042	0.920	1.181	1.041	0.852	1.270	0.932	0.787	1.103
	(0.066)			(0.106)			(0.080)		
30-34	0.983	0.850	1.136	0.816	0.643	1.035	1.202	0.987	1.464
	(0.073)			(0.099)			(0.121)		
35+	0.833	0.632	1.096	0.543*	0.335	0.882	1.335	0.907	1.964
	(0.117)			(0.134)			(0.263)		
Gender (ref.: Female)									
Male	0.930	0.842	1.027	0.923	0.785	1.086	1.021	0.893	1.169
	(0.047)			(0.077)			(0.070)		
Location of medical school (ref.: Ontario)									
West	0.961	0.783	1.180	0.565***	0.405	0.788	1.423*	1.081	1.872
	(0.101)			(0.096)			(0.199)		
Quebec	0.481***	0.386	0.598	0.465***	0.326	0.663	0.479***	0.358	0.641
	(0.054)			(0.084)			(0.071)		
East	0.837	0.679	1.030	0.562	0.401	0.789	1.123		
	(0.089)			(0.097)			(0.159)	0.851	1.481
Current year graduate (ref.: No)									
Yes	1.142	0.881	1.480	1.160	0.784	1.717	0.764	0.513	1.140
	(0.151)			0.232			0.156		
Constant	6.084***			1.752***			18.630***		
	(1.821)			(0.783)			(8.1722)		
Model fit statistics									
Log-likelihood		-5275.446			-1961.614			-2986.948	
McFadden's R^2^		0.119			0.256			0.104	
N		13449			5458			8041	

Statistical significance: *p < 0.05; **p < 0.01; ***p < 0.001 Standard errors are shown in parentheses. Ref. is reference category.

When analysis was stratified by the competitiveness of applicants’ first choice disciplines, the results from the overall sample are largely consistent with those observed in the sub-samples of competitive and less competitive programs (Table 2). However, in the competitive sample, matching success decreased significantly by 46% (OR = 0.543, *p* < 0.05) for applicants 35 years and older compared to younger applicants (26-29 years), and graduation from a medical school in Western Canada had a significant negative effect on matching success (OR = 0.565, *p* < 0.001). On the contrary, among applicants who chose less competitive disciplines as their first choice, the odds of matching success in the first iteration increased significantly by 42% for graduates from the West (OR = 1.423, *p* < 0.05). When the analysis was further stratified by year of the match (Tables 3a and 3b), we found the effects of number of applications submitted and number of rankings received by an applicant to be consistent and comparable to those presented in Table 2. For example, as Table 3a and 3b show, a higher number of rankings obtained by an applicant consistently predicted higher odds of matching to a first choice discipline in each of the seven years considered.

**Table 3a:** Logistic regression models examining the likelihood of matching to a first choice discipline in the first iteration by year

	Year: 2013			Year: 2014			Year: 2015			Year: 2016		
	OR	Confidence interval		OR	Confidence interval		OR	Confidence interval		OR	Confidence interval	
Number of applications submitted	0.915***	0.894	0.937	0.907***	0.888	0.926	0.906***	0.889	0.922	0.917***	0.901	0.933
Number of volunteering activities	0.978	0.945	1.012	1.019	0.988	1.050	1.001	0.971	1.032	0.985	0.960	1.012
Number of research activities	1.000	0.969	1.032	0.992	0.970	1.014	1.001	0.976	1.027	1.005	0.983	1.026
Number of publications	1.020	0.998	1.043	0.995	0.980	1.010	0.990	0.976	1.004	0.994	0.980	1.008
Number of program ranks received	1.247***	1.188	1.309	1.256***	1.201	1.314	1.250***	1.204	1.298	1.246***	1.202	1.292
Age (ref.: 26-29 years)												
< 25	0.790	0.545	1.144	1.235	0.844	1.805	0.963	0.676	1.373	1.311	0.933	1.844
30-34	0.767	0.483	1.218	1.200	0.783	1.841	0.867	0.571	1.318	1.060	0.727	1.546
35+	0.665	0.267	1.655	1.260	0.547	2.906	0.642	0.297	1.389	1.616	0.703	3.711
Gender (ref.: Female)												
Male	0.867	0.632	1.188	0.906	0.678	1.211	0.980	0.737	1.303	1.053	0.805	1.378
Location of medical school (ref.: Ontario)												
West	0.518	0.243	1.103	1.377	0.801	2.368	0.777	0.399	1.514	0.892	0.523	1.521
Quebec	0.358*	0.160	0.797	0.772	0.434	1.374	0.473*	0.232	0.964	0.686	0.384	1.227
East	0.439*	0.205	0.941	1.181	0.676	2.064	0.664	0.339	1.302	0.746	0.436	1.276
Current year graduate (ref.: No)												
Yes	1.277	0.576	2.831	0.694	0.297	1.621	1.266	0.628	2.549	1.035	0.497	2.155
Constant	7.257			7.448*			4.737			4.204		
Model fit statistics												
Log-likelihood		-561.598			-642.624			-656.533			-749.042	
McFadden's R^2^		0.119			0.122			0.154			0.131	
N		1656			1788			1737			1967	

Statistical significance: *p < 0.05; **p < 0.01; ***p < 0.001 Ref. is reference category. OR is odds ratio.

**Table 3b:** Logistic regression models examining the likelihood of matching to a first choice discipline in the first iteration by year

	Year: 2017			Year: 2018			Year: 2019		
	OR	Confidence interval		OR	Confidence interval		OR	Confidence interval	
Number of applications submitted	0.933***	0.920	0.946	0.928***	0.916	0.941	0.925***	0.912	0.939
Number of volunteering activities	0.982	0.959	1.006	0.992	0.968	1.017	0.993	0.975	1.011
Number of research activities	0.977*	0.959	0.996	0.972**	0.953	0.991	0.982*	0.967	0.997
Number of publications	1.004	0.989	1.018	0.998	0.986	1.009	1.010	1.000	1.021
Number of program ranks received	1.192***	1.157	1.228	1.147***	1.121	1.174	1.138***	1.112	1.166
Age (ref.: 26-29 years)									
< 25	0.902	0.658	1.236	1.3097	0.970	1.768	0.949	0.697	1.293
30-34	0.840	0.591	1.194	1.5388	1.058	2.238	0.714	0.502	1.015
35+	1.513	0.676	3.388	0.731	0.383	1.393	0.460	0.257	0.824
Gender (ref.: Female)									
Male	0.826	0.640	1.067	1.102	0.867	1.401	0.938	0.735	1.196
Location of medical school (ref.: Ontario)									
West	1.392	0.852	2.273	0.740	0.444	1.234	0.967	0.586	1.597
Quebec	0.550*	0.327	0.927	0.335***	0.195	0.575	0.402***	0.236	0.684
East	1.067	0.649	1.756	0.665	0.395	1.121	0.889	0.537	1.471
Current year graduate (ref.: No)									
Yes	1.202	0.625	2.310	1.537	0.821	2.878	1.015	0.536	1.919
Constant	3.633			3.771			9.278**		
Model fit statistics									
Log-likelihood		-803.251			-901.083			-874.090	
McFadden's R^2^		0.143			0.136			0.108	
N		2020			2124			2109	

Statistical significance: *p < 0.05; **p < 0.01; ***p < 0.001 Ref. is reference category. OR is odds ratio.

We also assessed the likelihood of matching to other outcomes including the possibility of matching to *any choice of discipline in the first iteration* only, and *any choice of discipline in both first and second iterations*. While the results are comparable to those presented in Table 2, some unique observations emerged. Table 4 reveals that compared with prior year applicants, current year graduates are 1.9 times significantly more likely (OR = 1.885, *p* < 0.01) to match to any discipline in both first and second iterations. Applicants aged < 25 years (OR = 0.756, *p* < 0.05) are less likely to match to any chosen discipline in both first and second iterations compared to applicants who are between 26 to 29 years old. Also, males appear to be significantly less likely to match to any choice of discipline in the first iteration (OR = 0.837, *p* < 0.05) as well as any choice of discipline in both first and second iterations (OR = 0.799, *p* < 0.05) compared to females (Table 4).

**Table 4:** Logistic regression models predicting other matching outcomes

	Overall sample			Overall sample		
	Any choice in first iteration			Any choice in first and second iteration		
	OR	Confidence interval		OR	Confidence interval	
Number of applications submitted	0.955***	0.946	0.964	0.976***	0.966	0.987
	(0.005)			(0.005)		
Number of volunteering activities	0.991	0.975	1.007	0.980*	0.962	0.999
	(0.008)			(0.010)		
Number of research activities	0.972***	0.961	0.983	0.965***	0.952	0.978
	(0.006)			(0.007)		
Number of publications	0.995	0.988	1.002	0.992	0.984	1.001
	(0.004)			(0.004)		
Number of program ranks received	1.699***	1.643	1.757	1.692***	1.621	1.766
	(0.029)			(0.037)		
Age (ref.: 26-29 years)						
< 25	1.044	0.849	1.284	0.756*	0.586	0.975
	(0.110)			(0.098)		
30-34	1.254	0.981	1.603	1.319	0.959	1.814
	(0.157)			(0.214)		
35+	1.434	0.944	2.179	1.318	0.793	2.193
	(0.306)			(0.342)		
Gender (ref.: Female)						
Male	0.837*	0.708	0.991	0.799*	0.644	0.991
	(0.072)			(0.088)		
Location of medical school (ref.: Ontario)						
West	0.448***	0.312	0.642	0.497**	0.311	0.792
	(0.082)			(0.118)		
Quebec	0.545**	0.374	0.794	0.628	0.388	1.018
	(0.105)			(0.155)		
East	0.434***	0.301	0.626	0.450***	0.281	0.722
	(0.081)			(0.108)		
Current year graduate (ref.: No)						
Yes	1.287	0.898	1.845	1.885**	1.274	2.789
	(0.237)			(0.377)		
Constant	2.038			1.892		
	(0.861)			(0.904)		
Model fit statistics						
Log-likelihood		-2055.686			-1360.881	
McFadden's R^2^		0.331			0.317	
N		13449			13449	

Statistical significance: *p < 0.05; **p < 0.01; ***p < 0.001 Standard errors are shown in parentheses. Ref. is reference category. OR is odds ratio.

## Discussion

This study examines factors influencing applicants’ likelihood of matching to their first choice or any discipline and assesses whether the effects of these vary by the competitiveness of a chosen discipline as well as the year of the match. Our results indicate that increasing the number of applications submitted is not associated with matching success. There seems to be no added benefit of submitting a higher number of applications in the first and second iterations for both competitive and less competitive disciplines. This is an important finding that is contrary to recent trends: the average number of CMG program applications in 2013 was 13.6 with a steady increase in the number of applications to 21.2 in 2019.^5^ The increase in applications over time may reflect students adopting a parallel planning strategy whereby applications are submitted to pursue more than one discipline. Students and career advisors might believe that submitting more applications increases the likelihood of matching success, however this finding shows that there is little, if any, benefit on matching outcomes. This has substantial implications for the overall matching process, which is resource and time intensive for both students and programs since each application requires file review followed by interview for some applicants. Fewer applications per candidate would likely ease the burden in processing candidate applications but also decrease the costs for applicants such as the application submission and travel for interviews. It is not entirely clear, however, which students might benefit from submitting fewer applications. Further research is much needed in this area.

The number of publications, volunteer, and research activities did not have a strong impact on the likelihood of matching in the first or second iteration, regardless of the discipline competitiveness. Similar to the number of submitted applications, there seems to be no added benefit of reporting more research activities. These results have profound implications for how medical students prepare for CaRMS during their medical school training. The purpose of pursuing activities such as research and volunteer activities is to encourage balance, altruism and scholarly development, which are considered important competencies for future medical doctors. There is a prevailing belief among students that enhancing one’s curriculum vitae with these items, and in particular research productivity, will increase the likelihood of a successful match.^20^ As a result, substantial time and effort is devoted to these activities that may increase medical student stress.^33^ The average self-reported number of volunteer activities was 7.9, research activities 8.2, and publications 8.6. The number of research activities for those pursuing a competitive discipline was higher than those for less competitive disciplines (8.8 vs. 7.7) and there was a notable increase in the numbers of reported research activities from 2013 - 2019. While these numbers may include activities completed prior to medical school, considerable efforts are still being devoted in that direction in the mistaken belief that they will strengthen the application.^18^^–^^22^ Within this highly competitive matching environment, these results suggest that there is no compelling justification to recommend increasing these activities for the sole purpose of improving match success.

The apparent disconnect between what medical students perceive to be important to match success and actual successful outcomes has previously been documented.^18^^,^^19^ Gupta et al. (2017) found that scholarly activity reported by applicants to Canadian Pediatrics residency programs was not associated with achieving a more desirable match outcome^19^. Furthermore, surveys of program directors in various programs have reported that research is considered to be of moderate or low importance when ranking an applicant.^20^^–^^22^ Our study corroborates these findings.

Applicant demographics played a significant role in matching outcomes. While age did not have an impact on matching in the first iteration, younger applicants (<25 years) were less likely to be matched after the second iteration. Males were significantly less likely to match in the first and second iterations. While prior studies report that the effect of gender on matching outcomes may vary by discipline type, our study did not examine discipline specific effects.^34^^,^^35^ Applicants from Ontario schools were significantly more likely to match in the first and second iterations of the match compared to all other schools. They also had a higher likelihood of matching to their first choice discipline compared to students from Western Canada or Quebec. Fortunately, efforts are currently underway to increase the number of postgraduate training positions as well as provide better supports for students who do not match.^36^ In 2018, for example, the Ontario government invested $23 million over six years to increase the number of residency positions for medical school graduates after initially eliminating existing positions in 2015.^36^^,^^37^ Current year CMGs were significantly more likely to match in the second iteration compared to prior year CMGs. These results demonstrate that demographic inequalities exist within the matching system that have not been previously highlighted. Special attention will be required to identify why these differences are present and how they can be addressed to ensure an equitable matching process.

To help address the causes and impact of unmatched medical students across Canada, we must first understand which students have a higher likelihood of going unmatched. We found that that unmatched students were exclusively from the pool of candidates that chose a more competitive discipline in first iteration. Having gone unmatched in a prior year of the match, being male, age <25, and having trained at a medical school in Eastern or Western Canada were all associated with a higher likelihood of going unmatched. This characterization of unmatched students has not been previously documented and may serve to focus attention on examining mitigating strategies and additional research.

## Limitations

One of the limitations of this study is that the residency match involves a number of subjective measures that were not available for examination. For instance, the quality of the personal letter, reference letters, and interview performance all play a role in matching outcome. In fact, surveys of program directors show consistently higher value placed on the interview, rotation evaluations, and expressed interest in the program above extracurricular, volunteer, and research activities.^21^^,^^22^ The amount of variance explained by the predictor variables in all the models estimated in this study is fairly low, ranging from approximately 10% to 33% (see Tables 2 to 4). Another limitation relates to the use of large sample size which allows small differences to be detected with statistical significance where the absolute difference may not be practically important or meaningful.^38^ For example, although the effect of research activities is statistically significant, the effect size observed is very small and is therefore unlikely to play much of a role in matching outcome. Finally, falsification of self-reported research activity, termed publication misrepresentation, has been reported in several studies of both Canadian and US candidates applying to surgical and non-surgical residency programs.^39^^–^^41^ The degree to which misrepresentation occurred in our sample could not be determined but has been reported to be as high as one quarter of applicants to a single surgical subspecialty.^40^

### Conclusion

The purpose of this study was to identify factors that lead to success in matching to a post-graduate training program. None of the application factors that could be modified by the applicant demonstrated an increased likelihood of successful matching. However, contrary to popular belief, more research activities reported, and more applications submitted were not associated with matching success. Given the heightened concern around the number of medical students going unmatched and the increased burden that this places on medical schools and future iterations of the match, it is essential that we analyze factors that may be contributing to this outcome, including biases within the selection process. Overall, we identified that those students who did not match at all had applied to competitive disciplines as their first choice disciplines, were more likely to be male, aged less than 25, and from Eastern and Western medical schools. The results of this study can help guide students and career advisors in developing appropriate application strategies, create awareness about factors associated with unsuccessful match outcomes and serve as a catalyst for further exploration into these factors.

## Appendix A

**Table 5:** Competitive and less competitive disciplines as defined by supply to demand ratios

Discipline Type	Supply to Demand Ratio Ratios < 1 were defined as competitive disciplines. Ratios > than 1 were defined as less competitive disciplines
Plastic Surgery	0.48
Dermatology	0.52
Emergency Medicine	0.60
Ophthalmology	0.69
Otolaryngology	0.70
Obstetrics / Gynecology	0.73
Urology	0.74
Anesthesia	0.76
Neurosurgery	0.80
Pediatrics	0.81
General Surgery	0.86
Physical Medicine and Rehabilitation	0.92
Vascular Surgery	0.94
Orthopedics	0.95
Diagnostic Radiology	0.96
Neurology	0.97
Cardiac Surgery	1.01
Psychiatry	1.05
Public Health + Family Medicine	1.09
Laboratomy Medicine	1.13
Pediatric Neurology	1.17
Radiation Oncology	1.27
Family Medicine	1.31
Medical Genetics	1.59
Anatomic Pathology	1.72
Nuclear Medicine	1.90
Public Health	2.24
Medical Microbiology	2.60
Neuro Pathology	2.88
General Pathology	3.40
